# Phenotyping the Structure and Function of the Heart of Elite Sailors: Implications for Pre-Participation Cardiac Screening

**DOI:** 10.3390/jcdd13010053

**Published:** 2026-01-20

**Authors:** Joseph D. Maxwell, Luca J. Howard, Ian White, Florence Place, Obipiseibima Aggokabo, Shaun Robinson, Camille S. L. Galloway, Jacob K. K. Shardey, Christian Verrinder, Keith P. George, Robert Cooper, David Oxborough

**Affiliations:** 1 Research Institute of Sports and Exercise Science, Liverpool John Moores University, Tom Reilly Building, Liverpool L3 3AF, UKd.l.oxborough@ljmu.ac.uk (D.O.); 2 Liverpool Centre for Cardiovascular Science, University of Liverpool, Liverpool, Liverpool John Moores University and Liverpool Heart & Chest Hospital, Liverpool L14 3PE, UK; 3 Liverpool University NHS Foundation Trust, Mount Vernon Street, Liverpool L7 8XP, UK; 4 British Sailing Team, Performance Unit, Portland, Liverpool DT5 1FA, UK; 5 Imperial College Healthcare NHS, London W2 1NY, UK; 6 School of Health and Exercise Sciences, University of British Columbia, Okanagan, Kelowna, BC V1V 1V7, Canada; 7 Liverpool Heart and Chest Hospital, Liverpool L14 3PE, UK

**Keywords:** athletes’ heart, pre-participation cardiac screening, cardiac remodelling, sailing, echocardiography

## Abstract

*Background:* Structural and functional adaptation of the heart to chronic exercise is dependent on multiple factors, including the volume and type of training, and has direct implications for pre-participation cardiac screening (PPCS). Sailing is a unique multi-training modality sport with limited prior description of cardiac adaptation to training. The aims of this study are (1) to describe electrocardiogram (ECG) changes in sailors, informing PPCS guidelines; (2) to assess structural and functional cardiac changes in sailors; and (3) to examine sex- or discipline-specific cardiac adaptations in sailors. *Methods:* Seventy elite sailors (33 females) underwent standard ECG and echocardiography. Echocardiographic data were compared to population norms and analysed by sex and sailing discipline based on training type: isometric (IG), pumping (PG), and movement (MG). *Results*: One sailor presented with abnormal ECG findings (T wave inversion) which warranted further investigation. Primary training-related ECG changes noted were early repolarisation (24%) and sinus bradycardia (30%). The left ventricular volume index was dilated in 18% of all sailors compared to reference values, with similar findings noted on right ventricular parameters for 22% of the study population, although in males only. The impact of predominant training stimulus (IG, PG, MG) did not mediate differences in the structure of any cardiac chambers (*p* > 0.05). Ejection fraction was lower in the PG (Δ4%, *p* ≤ 0.001), whereas global longitudinal strain was higher (Δ2%, *p* = 0.02) compared to MG and IG. *Conclusions*: Elite-level sailors present with electrical and structural cardiac phenotypes associated with exercise adaptation, with dilation of both left- and right-sided chambers. These data should be considered when interpreting results of PPCS in male and female sailors from different, specific disciplines.

## 1. Introduction

Chronic exercise training promotes electrical, morphological, and functional cardiac adaptation, termed ‘the athlete’s heart’ [1,2]. These adaptations are heterogeneous, and can vary significantly due to sex, age, ethnicity, as well as training type and volume [1,2,3]. Increased chamber size (both ventricular and atrial), increased wall thickness, and low resting function are frequently observed in athletic populations alongside electrical adaptations [4,5]. Indeed, it is such exercise-induced adaptations which represent a major diagnostic challenge during pre-participation cardiac screening (PPCS). Sudden cardiac death (SCD) is the leading cause of mortality in young athletes [6], and PPCS using 12-lead electrocardiogram (ECG), often in conjunction with transthoracic echocardiography, is widely used to assess for underlying cardiac conditions [7,8].

Understanding the acute physiological stimulus (i.e., training type) which underpins cardiac adaptations allows for more nuanced assessments of both ECG and echocardiography data. Such information is fundamental in providing guidance for the growing body of sporting organisations who mandate PPCS. Historically, strength-based sports were associated with left ventricular (LV) hypertrophy, and endurance-based sports with LV dilation [9]. In more recent years, numerous studies have challenged this concept [10,11]. Nevertheless, the hypothetical assumption of exercise-specific cardiac adaptations is primarily centred around haemodynamic volume (associated with endurance exercise) and pressure (with resistance exercise) overload. However, such simplistic haemodynamics to training are less binary, given that a vast array of sporting disciplines require significant mixed stimuli (pressure and volume), alongside isotonic and isometric components [12].

Sailing is a sporting discipline with a varied competition structure and mixed-training stimuli. The sport requires substantial full-body muscular endurance, strength, power, as well as both anaerobic and aerobic capacity [13,14]. Although there are nuances within the discipline (i.e., single/double dinghy, windsurf, and kitesurf, with subvariants of each discipline), overall, there is a moderate-high physiological load, with athletes achieving 40–77% of VO_2max_ and 74–87% of HR_max_ during simulated competition [15]. Insights into cardiac adaptions in sailing disciplines are extremely limited and those that have been carried out are largely outdated and employed basic LV assessment metrics to demonstrate larger LV volume and mass when compared to sedentary age-matched controls [16]. These data do not highlight the holistic cardiac phenotype of an elite sailor (electrical or structural) and fail to acknowledge any variance due to factors such as sex and sailing disciplines. Therefore, the aims of the study are (1) to describe electrocardiogram (ECG) changes in sailors, which could inform PPCS guidelines, (2) assess structural and functional cardiac changes in sailors and (3) examine sex- or discipline-specific cardiac adaptations in sailors.

## 2. Methods

### 2.1. Study Population and Design

Seventy elite sailors (male *n* = 37 and female *n* = 33; all Caucasian ethnicity) were recruited during their PPCS as a part of the British national sailing team. Participants completed a questionnaire, documenting any current cardiovascular symptoms, and family history of cardiovascular disease. All clinical data were analysed and reported by a Sports Cardiologist to exclude any underlying cardiac disease. Sailing discipline (Laser standard, 470 Helm, Kiteboarding, 470 Crew, Windsurfing, 49er Crew, 49er Helm and Nacra 17), training history and current training volume/duration were reported. Ethics approval was obtained from the Health Research Authority (IRAS project ID: 169429) and athletes provided full written informed consent to participate in the study.

### 2.2. Procedures

All participants abstained from exercise training, alcohol, and caffeine for at least 24 h prior to data collection and were non-smokers. Height (Seca 217, Hannover, Germany) and weight (Seca supra 71, Hannover, Germany) were documented and body surface area (BSA) was calculated as previously described [17]. Resting arterial blood pressure (BP) was measured using an automated blood pressure machine (Dinamap Carescape V100, GE Healthcare, Buckinghamshire, UK). A standard 12-lead ECG was acquired (SECA CardioPad2, Birmingham, UK) and interpreted using the current “international criteria” for the athletes ECG by a Sports Cardiologist [12].

#### 2.2.1. Echocardiographic Measurements

All echocardiographic data were collected by three clinically accredited sonographers (JDM, SR and DO) using commercially available ultrasound systems (Vivid IQ, GE Healthcare, Horten, Norway) with 1.5–4.0 Mhz phased array transducer. Images were acquired with the participant lying in the left lateral decubitus position and stored in raw Digital Imaging and Communications in Medicine before being exported to an offline workstation (EchoPAC version 204; GE Healthcare) for analysis. All echocardiographic data were acquired in adherence to published British Society of Echocardiography (BSE) guidelines [18].

#### 2.2.2. Conventional Measurements

All measurements were made in accordance with BSE Guidelines [18]. The following left heart indices were assessed: LV internal dimension at end diastole (LVIDd) and end systole (LVIDs). End diastolic LV wall thickness was measured at four locations (infero-septum, anteroseptum, inferolateral, and anterolateral) at both the basal and mid-cavity level obtained from parasternal short axis (PSAX) orientations and averaged to produce mean wall thickness (MWT) [7]. Relative wall thickness (RWT) was calculated according to the following formula: (basal anterior wall thickness + basal inferolateral wall thickness)/LVIDd. LV mass was determined using the American Society of Echocardiography corrected equation and LV geometry was categorised based on a combination of LV mass indexed (to BSA) and RWT [18]. LV end-diastolic (LVEDV) and systolic volume (LVESV) were calculated using Simpson’s biplane method and LV ejection fraction (LVEF) was calculated. Tissue Doppler imaging of the septal and lateral mitral annulus (LV s’, e’, a’), as well as transmitral Doppler (E, A and E/A ratio) were obtained. Left atrial volume (bi-plane method) was measured at end systole (LAESV). Regarding the right heart, end-diastolic linear measurements were made at the inflow base (RVD1), mid (RVD2), proximal outflow (RVOT1), and distal outflow (RVOT2). The RV end-diastolic (RVED) area and the RV end-systolic (RVES) area were measured and the RV fractional area change (FAC) calculated. Tissue Doppler imaging of tricuspid annulus (RV s’, e’, and a’), tricuspid annular plane systolic excursion (TAPSE), and right atrial (RA) end-systolic area were also obtained. Structural indices were scaled linearly to BSA for comparison to published normal ranges for non-athletes. To allow comparisons between groups and sexes, indices were also allometrically scaled to BSA based on the principle of geometric similarity [19], i.e., linear dimensions were scaled to BSA^0.5^, volumes to BSA^1.5^, and areas directly to BSA.

#### 2.2.3. Myocardial Strain Imaging

**Figure 1:**
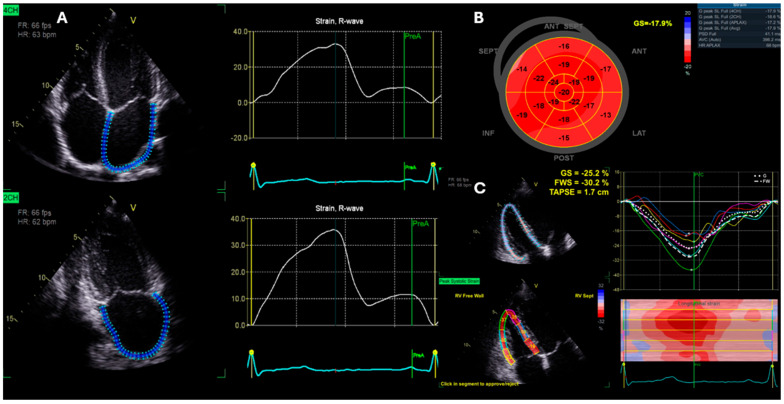
Example of Speckle tracking echocardiography for the assessment of left atrial reservoir, conduit and booster strain (**A**), global longitudinal left ventricular strain bullseye (**B**) and right ventricular free wall strain (**C**).

Cardiac mechanics were assessed by Speckle tracking echocardiography. Images were acquired with frame rates between 40–90 frame/s, with depth and sector widths optimised to ensure adequate imaging of the chamber of interest and ensure clear endocardial definition. The apical four-chamber, two-chamber, and long-axis views were used for LV global longitudinal strain (GLS), whilst the RV-focused view was used for RV free wall strain (RVFWS) (Figure 1). Closure times for systole were achieved using event markers on aortic and pulmonary valve closure clicks using pulsed-wave Doppler signals. For LV GLS, an average of all wall segments from apical views was used to generate a single GLS value. For RVFWS, an average of three free wall segments was used [20,21]. If inappropriate tracking of segments was observed visually or detected by EchoPac, retracing was performed until all segments were considered acceptable or excluded from analysis if tracking was not possible on two or more segments. In addition, speckle tracking of the left atrium (LA) was carried out to obtain LA reservoir (maximal filling), conduit (passive filling), and booster (active emptying) longitudinal strain from LA optimized views in apical four- and two-chamber views (Figure 1). An average of six atrial segments was calculated to give a single value for reservoir, conduit and booster strain. All data were analysed by a single BSE-accredited echocardiographer.

#### 2.2.4. Sailing Groups

To investigate discipline-specific adaptations, sailors were divided into groups based on the physiological load and training type associated with each of the individual disciplines. These categories were selected by professional exercise physiologists with extensive sailing training and performance experience. The groups were as followed: isometric [IG] (laser standard, 470 helm, and kiteboarding), pumping [PG] (470 crew and windsurfers), and movement [MG] (49er crew, 49er helm, and nacra 17). Sailors within IG create boat/board speed through quasi-isometric force, predominantly through knee extension. Sailors in the PG create boat/board speed through a dynamic, rhythmic pull and push of the boom or trapeze wire so that the sail acts as a beating wing, providing additional propulsion, whilst the MG sailors are fast double handed boats which are required to move around the boat to optimise manoeuvres and straight-line speed.

#### 2.2.5. Statistical Analysis

All study data collected were stored and managed using REDCAP electronic data capture tool hosted by University College London. All echocardiographic indices for male and female sailors were assessed for normal distribution using a Kolmogorov–Smirnov test and presented as mean ± SD; the percentage of athletes that exceeded the normative range for non-athletes [22] are presented. To establish mean differences among IG, PG, and MG, a two-factor (Group*Sex) ANOVA with post-hoc Bonferroni adjustment for the assessment of between-group difference was undertaken. Statistical significance was accepted at *p* < 0.05. Statistical analysis was performed using a commercially available software package, i.e., SPSS Version 29.0 for Windows (SPSS, Chicago, IL, USA).

## 3. Results

### 3.1. Participant Characteristics

Participant characteristics for all sailors (based on sex and training groups) are displayed in Table 1. There was a main effect of sex for weight, height, body surface area, and systolic blood pressure, with such an effect being greater (*p* ≤ 0.05) in male sailors. There was a main effect of group for weight (*p =* 0.03), with PG sailors being heavier (*p* = 0.01) than MG sailors (Table 1).

#### ECG Parameters

Continuous ECG parameters are presented in Table 2, and the prevalence of training-related ECG changes are presented in Table 3. Sinus bradycardia was present in 32% of male and 27% of female sailors. Early repolarisation was noted in 32% of male and 15% of female ECGs. Increased voltage QRS criteria were observed in 22% of male ECGs and only in 3% of female ECGs, with sinus arrhythmia detected in 11% of male and 12% of female sailors (Table 3).

### 3.2. Left Ventricular Parameters

**Figure 2:**
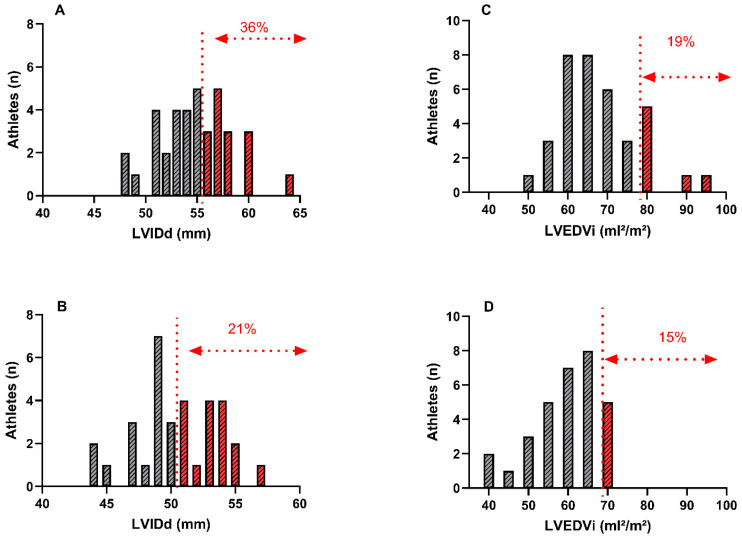
Range of values for LV internal dimension in diastole (LVIDd) and LV end diastolic volume, indexed to body surface area (LVEDVi), for male (**A**,**C**) and female (**B**,**D**) athletes. Red dotted lines represent cut-off for abnormal size based on British Society of Echocardiography reference intervals.

**Figure 3:**
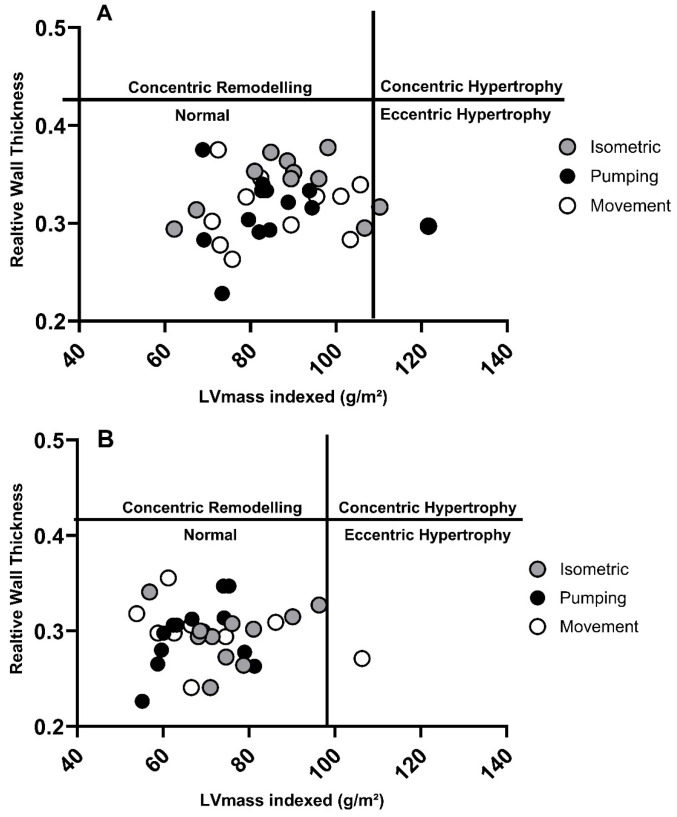
Left ventricular geometry data for male (**A**) and female (**B**) athletes.

In male sailors, 36% had an LVIDd which exceeded normal BSE reference values, with 19% above reference values for indexed LVEDV (LVEDVi). Similarly, 21% of female sailors had a LVIDd which was greater than normative values, and 15% of females exceeded LVEDVi values (Table 4 and Figure 2). Only 8% of male athletes and 6% of female athletes had an indexed LV mass which fell outside the normative range, and no athletes presented with a RWT that exceeded reference values (Table 4). One male and one female athlete demonstrated eccentric remodelling, with all other athletes having normal LV geometry (Figure 3). 38% of male athletes presented with an EF which was below the BSE normal range, whilst only 9% of females were lower (Table 5). All metrics of diastolic function assessed were within normative ranges.

**Figure 4:**
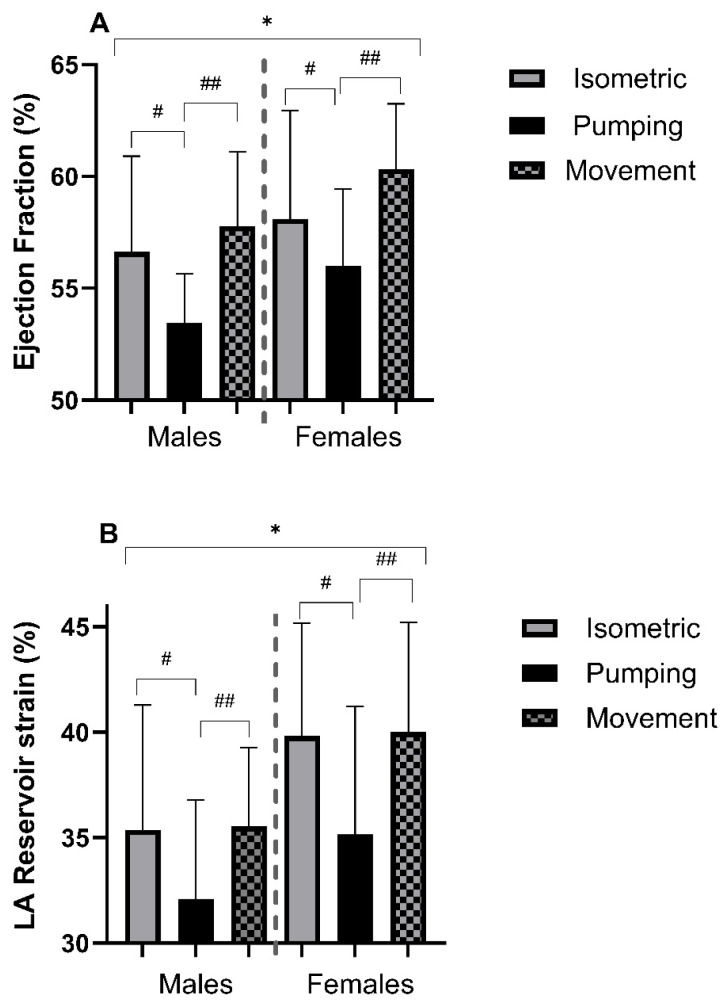
Left ventricular ejection fraction (**A**) and left atrial reservoir strain (**B**). * Denotes a significant difference (*p* < 0.05) between sexes. # Denotes a significant (*p* < 0.05) difference between isometric and pumping groups. ## Denotes a significant (*p* < 0.05) difference between pumping and movement groups.

There were significant main effects of sex (*p* ≤ 0.05) on all structural LV parameters, including absolute and scaled parameters, with higher values in males and no main effects of group (Table 4). There was a significant main effect of sex on EF (*p* = 0.01), with females having a higher EF (Table 5). There was also a significant main effect of group (*p* ≤ 0.001), with the PG sailors having a lower EF than that in either the IG or MG group (*p* = 0.04 and *p* ≤ 0.001, respectively, Figure 4). GLS was higher in females (*p* = 0.04), with a main effect of group (*p* = 0.02); sailors in the PG group had a significantly higher GLS compared to those in the MG group (*p* = 0.03, Table 5).

### 3.3. Right Ventricular Parameters

A total of 22% of all males had an indexed RVED area greater than reference values, with only 6% of female athletes above this cut-off value. In addition, 14% of males and 9% of females presented with an RVD1 base which was greater than normal values (Table 6). There was a main effect of sex across all RV structural parameters, absolute and indexed, with greater dimensions in male sailors (*p* ≤ 0.05, Table 6). There were no main effects of sex, group, or interactions for any RV functional parameter (*p* > 0.05, Table 6).

### 3.4. Atrial Parameters

Only 3% of males and females displayed indexed LA ESV volumes greater than normal reference ranges (Table 7). No male athlete presented with any LA strain parameter outside the normal reference range, whilst 3% of females displayed LA reservoir strain below normal values (Table 7). In male athletes, 16% and 8% had greater absolute RA area and indexed RA area, respectively, compared to reference values. Similarly, for female athletes, 6% presented with absolute RA size above normal reference limits, but these data were normalised when indexed to BSA.

Males had a greater LAESV (*p* ≤ 0.001) compared to females, however there was no sex difference when indexed (*p* ≥ 0.05, Table 7). Additionally, male sailors had larger RA areas, both absolute and scaled (*p* ≤ 0.001, *p* = 0.01) compared to females. There was no effect of group on any structural atrial measurement (*p* ≥ 0.05, Table 7). Female sailors had greater reservoir strain (*p* = 0.02) and lower pump strain (*p* = 0.01) compared to males. Sailors in the PG had a lower reservoir strain compared to both IG (*p* = 0.03) and MG (*p* = 0.04) groups, with no significant sex-group interaction observed (*p* = 0.86, Figure 4). Furthermore, there was a significant main effect of group (*p* = 0.002) for LA conduit strain, with the PG sailors having a lower conduit strain compared to the IG (*p* = 0.002) and the MG groups (*p* = 0.005, Table 7).

## 4. Discussion

The central aims of this study were to investigate the electrical, structural and functional cardiac phenotype of the elite sailor, outlining the prevalence of cardiac adaptations which present outside of the ‘normal range’ to better inform future PPCS. Our secondary aim was to explore whether cardiac phenotype differed between sex and sailing disciplines. The main findings of this study were that (i) sinus bradycardia and early repolarization are the most commonly observed training-related ECG findings in elite sailors; (ii) in ~20% of sailors, LV size would be categorised as abnormal and dilated even when indexed to BSA; (iii) similar findings for RV structural data were present in males only; and (iv) some left-sided functional parameters (LVEF, GLS, and LA reservoir strain) did differ between disciplines, with lower function noted in the PG.

### 4.1. The Sailor’s Heart

The resting 12-lead ECG represents the primary first line investigation for PPCS in the detection of underlying/inherited cardiac conditions [12]. Current understanding of ‘normal’ training-induced ECG changes largely stems from studies involving large athletic cohorts, with limited attention given to the unique cardiovascular demands of specific sports. Whilst there has been a shift in focus to examining endurance-based athletes vs. non-endurance-based athletes [23], sport-specific ECG data, which can, in turn, form a crucial part of guidance for PPCS policy, is still lacking. Our ECG data demonstrate training related ECG changes in elite sailors, with a predominance towards sinus bradycardia, early repolarisation, sinus arrhythmia, and increased QRS voltage criteria. In line with international guidance on athlete ECG interpretation, these do not warrant further investigation in the asymptomatic athlete [12].

An increase in LV chamber dimensions secondary to aerobic/isotonic training is a common finding irrespective of imaging modality [2,7,8,9,10]. Such adaptations are largely mediated by frequent and sustained increases in cardiac output (CO) with reduced or normal peripheral vascular resistance [5,24,25,26]. Sailing is a complex sport with a mixed-training approach. Depending upon the specific discipline, athletes balance their training across resistance training, aerobic training, and actual sailing time; with regard to the sailing time, the physical demands combine aerobic, isometric, concentric, and eccentric activity. This is, then, further complicated by the significant technical and tactical component of the sport. Indeed, the very nature of sailing frequently is classified as a *skill*-based sport, as opposed to endurance- or power-based sports. Nevertheless, ~20% of sailors presented with LVEDVi above normal values, which is unsurprising given the broad range of cardio-respiratory fitness amongst sailors. Data from previous work have outlined VO_2max_ values of elite sailors ranging from 50–65 mL/min/kg [27,28,29], with the broad range likely reflecting the different disciplines. Unfortunately, in the present study we were unable to obtain recent VO_2max_ data to identify if the athletes with the higher aerobic capacity did indeed present with a greater LVEDVi. From a PPCS perspective, a dilated LV presents as a diagnostic challenge in differentiating exercise-induced adaptations vs. underlying pathology (i.e., dilated cardiomyopathy). However, based on our data of prevalence, sailors presenting with dilated LVd should be interpreted alongside athletics-specific guidelines and only warrant further investigation based on clinical context or functional abnormalities [7].

Comparisons between previous studies are limited. Work by Meloni, Bonomo, Cherchi [11,16] showed that windsurfers (PG) had increased LVIDd, wall thickness and LV mass compared to controls. Whilst our data are in partial agreement with an increased LV dimension, our data on wall thickness fall within the normative range, disputing the claim that the prevalent isometric work component during windsurfing induced some form of LV hypertrophy. It is highly likely that the enhanced image resolution of modern 2D echocardiography is far superior to the M-mode techniques utilised by Meloni, Bonomo, Cherchi [11]. Our data regarding LV geometry, with only two athletes presenting with eccentric hypertrophy and all other athletes having normal geometry, are consistent with the most recent evidence research, especially in Caucasian athletes [30], and further supports the notion that athletes demonstrating concentric hypertrophy likely warrant further investigation for potential pathology in the context of PPCS [7].

Exercise-induced cardiac remodelling is not just limited to the LV, as a growing body of research has highlighted structural and functional RV changes [3]. We observed 22% of male athletes presenting with increased RV dimensions (RVED area indexed), whereas only 6% of females had dilatation above normal values. Sports such as sailing, which is classically described as a *skill* or *technical* sporting type have typically been associated with smaller RV dimensions when compared to power-based or endurance sports [31]. Like the LV, sports associated with higher and more prolonged exposure to elevated cardiac outputs and thus greater filling, are likely to demonstrate the larger RV dimensions. Unlike the LV and the downstream systemic circulation, the pulmonary circulation functions in a low resistance/high compliance state, therefore during exercise the pulmonary circulation has very limited ability to adjust for the rise in stroke volume. It is likely, therefore, that the rise in pulmonary pressures during sailing are modest when compared to endurance-based sports (distance running and cycling), with no athletes presenting with RVOT dimensions above normal limits, and 22% of male athletes with increased RV area, compared to 40% and 57% in endurance-based athletes [32]. Nevertheless, >20% of male sailors presenting with increased RV dimensions is of clinical significance, largely due to RV dilation being a feature in arrhythmogenic right ventricular cardiomyopathy (ARVC/AVC) [33]. Approximately 14% of SCD incidences are attributed to ARVC/AVC [34], therefore clear differentiation of exercise-induced RV remodelling from underlying pathology is essential in order to avoid false positives resulting in unnecessary and costly further investigations.

Increased LA volume in athletes is a consistent finding [35], and in the absence of diastolic dysfunction and/or significant valve disease, should be interpreted as physiological adaptation [7]. Aerobic exercise induces significant atrial volume overload secondary to increases in stroke volume/venous return [5], thereby endurance athletes tend to present with atrial dilation [36]. Given the training nature of elite sailors, whereby the primary training focus is predominantly on sailing rather than specific aerobic and/or resistance training, the 3% of athletes with *dilated* atria (>38 mL/m^2^) compared to normal reference values is unsurprising, and consistent with previous large-scale studies [37]. Additionally, we observed a slightly higher percentage (8%) of males with ‘dilated’ RA area indexed to BSA (≥11 cm^2^/m^2^). Due to its thinner walls, the right heart is known to be particularly sensitive to volume overload and similar to other cardiac chambers the greater RA dimensions are associated more with endurance athletes [38,39].

### 4.2. Impact of Sex

Left ventricular structural data, absolute and indexed, are greater in male sailors. Such findings align with available literature [40]. Interestingly, 20–30% of sailors (male and female) had LV measures above normal limits. Such observations are consistent with previous work stating that whilst both males and females exhibit similar adaption patterns, males tend to be greater in magnitude [1,41]. Underlying mechanisms explaining sex differences are speculative, but may relate to increased levels of testosterone, a higher density of myocardial androgen receptors in males or even simply different cardiac load mechanics with higher BPs at rest and during exercise in males [42,43]. Functionally, we observed a small but significant difference in EF and GLS, with females having increased function. Such observations are in agreement with previous research [12,13,14,15] and are likely a consequence of smaller LV cavity sizes.

The extent in which sex alters an individual’s cardiac remodelling is relevant as PPCS becomes more prevalent for elite female athletes [44,45,46,47]. There is some evidence to suggest that male athletes demonstrate a more profound RV adaptation to exercise than females [48,49], which aligns with our dataset with RV dilation noted in 22% of males and just 6% of females. Additionally, we observed statistically significant differences in all RV structural measurements, with males having larger dimensions in absolute and scaled (BSA and allometrically) parameters, yet no RV functional differences were noted between sexes. Our data from scaled RV parameters conflict with that of D’Ascenzi, Pisicchio, Caselli, Di Paolo, Spataro, Pelliccia [16], who identified that BSA indexed RV dimensions were relatively larger in females. One likely explanation for differences in findings may be the athlete cohort examined, whilst we solely focused on sailors (skill-based), the work by D’Ascenzi, Pisicchio, Caselli, Di Paolo, Spataro, Pelliccia [16] examined skill-, power-, mixed-, and endurance-based athletes. The interaction between sex and cardiac remodelling is likely complex and multifactorial. An elegantly designed study by Howden, Perhonen, Peshock, Zhang, Arbab-Zadeh, Adams-Huet, Levine [50] outlined that the time course of RV adaptation may differ between sexes following a 1-year endurance training intervention, with a delayed RV response in female athletes. The underlying mechanism driving these sex differences certainly warrants further investigation.

### 4.3. Impact of Sailing Discipline

Comparison of the sailor’s heart phenotype to that of other sporting adaptations is highly complex due to the heterogeneous nature of the sport with its disciplines. Within sailing, despite some differences, albeit small, in training-specific haemodynamic loading, we did not observe any difference between groups in structural LV parameters. One possible explanation may be related to the relatively small magnitude of training variation between groups. We did, however, note statically significant differences in LVEF between groups, with lower EFs noted in the PG. Given that athletes can present with reductions in EF, often driven by changes in LVEDV as physiologically enlarged chambers require a lower contractile state in order to maintain adequate stroke volume [51], our findings of differences in EF, but not LVEDV, are surprising. Similarly, we observed no significant difference in baseline haemodynamics (HR and BP) which can significantly alter EF. Interestingly, we simultaneously observed a higher GLS in the PG, suggestive of reduced longitudinal shortening/deformation of the myocardium. Since LV contraction is driven by longitudinal, radial and circumferential shortening, it is possible that sailors in the PG had increased radial/circumferential strain to compensate for reductions in GLS with some evidence suggesting altered strain mechanics between sports [52]. Whether actual LV contractility differed between groups, we can only speculate, and the associated LV mechanics are beyond the original scope of our aims, however future work assessing subtle differences in ventricular function should look to explore additional strain and strain rate parameters by means of 2D and 3D echocardiography.

Interestingly, despite no structural differences between sailing groups, we did observe a significantly lower LA reservoir strain in the PG sailors. Reservoir strain is a marker of atrial compliance and reflects the atrial pressure-volume relationship [53,54]. Previous work identified an inverse relationship between reservoir strain and ‘cardiac demand’, with those athletes who participated in lower intensity sports (skill-based) having the highest reservoir strain values [55]. Our dataset shows conflicting results to this, with the PG having the lowest reservoir strain. In contrast, one study observed no difference in atrial strain in high vs. low dynamic sporting disciplines and healthy controls, whereas differences in functional volumes (passive and active emptying volumes) was significant between groups [56]. A meta-analysis of 403 elite-level athletes (variety of sporting disciplines) showed reduced reservoir strain in athletes compared to healthy controls [35], which is inconsistent with our findings of only 3% of athletes having a reservoir strain outside the normative range, further emphasizing the impact of different sporting types, and the underpinning physiology, on adaptation [5].

### 4.4. Limitations

Whilst we present data from similar numbers of male and female sailors, our data are limited exclusively to Caucasian athletes, and it is important to acknowledge the role of ethnicity within exercise-induced cardiac adaptation [5,57]. As previously mentioned, within our dataset we were unable to present VO_2max_ or muscular strength data, which would have provided an interesting insight into overall cardiorespiratory fitness of the athletes being assessed, and whether VO_2max_ differed between groups, given the strong association between VO_2max_ and cardiac remodelling [58]. Finally, whilst our categorisation of the different sailing disciplines into three individual groups was based on the physiological stimulus propelling the boat, with a larger sample size, examining and comparing the individual sailing disciplines may have revealed more subtle differences. Whilst all data were collected and analysed using commercially available software, all measurements were performed using a single software and therefore may not be directly comparable to those of other vendor and/or models. This is particularly apparent with GLS data, whereby each vendor uses proprietary algorithms for strain calculations. Ultimately, the minimal differences in cardiac parameters may reflect small differences in athletic workload alongside relatively small populations within each group. Quantifying the specific workload of sailors from different disciplines is incredibly challenging due to the complex nature of the sport being water-based. A larger sample size would allow for interrogation of cardiac adaptations based on individuals sailing discipline, rather than the ‘grouping’ approach taken in this study. By doing so, this may uncover more meaningful discipline-specific adaptations.

## 5. Conclusions

The significant variation in sailing disciplines and techniques alongside its large skill component make it a fascinating model of assessing physiological adaptation. We have shown typical training-related ECG changes occur in 15–20% of elite-level sailors and elite sailors can present with exercise-induced cardiac adaptation with dilation of both left- and right-sided chambers, independent of sailing discipline, whereas functionally, 470 crew and windsurfers may present with ‘lower’ parameters of LV function. Additionally, we have outlined that female sailors also present with athlete heart characteristics, although to a lesser extent than males. These data should be considered when interpreting results for PPCS in elite sailing.

## Figures and Tables

**Table 1:** Participant characteristics.

*Parameter*	*All Sailors*	*Isometric Group (IG)*	*Pumping Group (PG)*	*Movement Group (MG)*
**Males**				
*Samples Size*	*37*	*11*	*13*	*13*
Age (years)	24 ± 5	23 ± 4	22 ± 4	26 ± 6
Weight (kg) *	84 ± 9 **	87 ± 7	87 ± 9	82 ± 5 **^##^**
Height (cm) *	183 ± 6	185 ± 5	184 ± 6	182 ± 5
Body surface area (m^2^) *	2.06 ± 0.13	2.11 ± 0.11	2.10 ± 0.13	2.03 ± 0.11
Heart rate (bpm)	62 ± 10	63 ± 11	63 ± 7	57 ± 8
Systolic blood pressure (mmHg) *	124 ± 10	125 ± 7	122 ± 9	126 ± 11
Diastolic blood pressure (mmHg)	73 ± 8	73 ± 9	74 ± 9	73 ± 7
Training hours (per week)	17 ± 5	17 ± 7	18 ± 7	17 ± 4
**Females**				
*Samples Size*	*33*	*11*	*13*	*9*
Age (years)	24 ± 4	24 ± 4	23 ± 3	24 ± 4
Weight (kg) *	70 ± 6 **	69 ± 6	72 ± 6	67 ± 7 **^##^**
Height (cm) *	171 ± 7	172 ± 8	171 ± 6	169 ± 7
Body surface area (m^2^) *	1.82 ± 0.10	1.82 ± 0.11	1.85 ± 0.08	1.77 ± 0.11
Heart rate (bpm)	64 ± 10	62 ± 11	63 ± 9	65 ± 11
Systolic blood pressure (mmHg) *	114 ± 7	114 ± 8	112 ± 9	116 ± 3
Diastolic blood pressure (mmHg)	71 ± 9	70 ± 11	73 ± 8	70 ± 8
Training hours (per week)	18 ± 3	19 ± 4	17 ± 3	17 ± 3

* Denotes main effect of sex. ** Denotes main effect of group. **^##^** Denotes a significant difference (*p* < 0.05) between PG and MG.

**Table 2:** Continuous ECG parameters for sailors.

*ECG Parameter*	*Males (n = 37)*	*Females (n = 33)*
HR (bpm)	62 ± 10	64 ± 10
P wave duration (ms)	106 ± 10	101 ± 10
PR interval (ms)	161 ± 25	150 ± 19
QRS duration (ms)	110 ± 49	88 ± 10
QTc interval (ms)	402 ± 19	419 ± 22
P axis (°)	41 ± 16	40 ± 20
QRS axis (°)	56 ± 24	53 ± 19
T axis (°)	28 ± 13	34 ± 13
Voltage criteria for LVH (mm)	31 ± 8	25 ± 7
Voltage criteria for RVH (mm)	7 ± 3	4 ± 2

**Table 3:** Prevalence of normal training-related ECG findings in Sailors.

*ECG Finding*	*Males (n = 37)*	*Females (n = 33)*
Increased QRS voltage criteria	8 (22)	1 (3)
Incomplete RBBB	0 (0)	0 (0)
Early repolarisation	12 (32)	5 (15)
Sinus bradycardia	12 (32)	9 (27)
Sinus arrhythmia	4 (11)	4 (12)
Ectopic atrial rhythm	0 (0)	0 (0)
Junctional escape rhythm	0 (0)	0 (0)
First degree AV block	1 (3)	0 (0)
Mobitz type I (Wenckebach) second degree AV block	0 (0)	0 (0)

Data presented as *n* (%) of sailors. Abbreviations: RBBB (right bundle branch block) and AV (atrioventricular).

**Table 4:** Left ventricular structural parameters.

*Parameter*	*Mean ± SD of All Sailors (Range)*	*BSE Normative Range*	*% of Athletes Outside Normative Range*	*IG (Mean ± SD)*	*PG* *(Mean ± SD)*	*MG* *(Mean ± SD)*
**Male (*n* = 37)**						
LVIDd (mm) *	55 ± 4 (48–64)	37–56	36%	54 ± 3	56 ± 4	55 ± 4
LVIDd index (mm/(m^2^)^0.5^) *	38 ± 3 (33–45)	n/a	n/a	37 ± 2	38 ± 3	39 ± 3
LVEDV (mL) *	146 ± 22 (107–209)	53–156	27%	151 ± 19	143 ± 20	149 ± 27
LVEDV index (mL/m^2^) *	70 ± 10 (52–97)	30–79	19%	50 ± 7	47 ± 6	52 ± 8
LVEDV index (mL/(m^2^)^1.5^) *	50 ± 10 (52–97)	n/a	n/a	50 ± 7	47 ± 6	52 ± 8
LV mass (g) *	178 ± 30 (128–257)	72–129	8%	187 ± 32	179 ± 33	180 ± 33
LV mass (g/m^2^) *	86 ± 13 (62–121)	40–110	5%	89 ± 15	85 ± 14	88 ± 14
Mean wall thickness (mm) *	9 ± 1 (8–10)	6–12	0%	8.8 ± 0.4	8.5 ± 0.6	8.4 ± 0.6
Relative wall thickness *	0.32 ± 0.04 (0.27–0.38)	≤0.42	0%	0.34 ± 0.03	0.32 ± 0.03	0.32 ± 0.03
**Female (*n* = 33)**						
LVIDd (mm) *	50 ± 3 (44–57)	35–51	21%	52 ± 3	50 ± 3	49 ± 3
LVIDd index (mm/(m^2^)^0.5^) *	38 ± 3 (33–45)	n/a	n/a	39 ± 2	37 ± 2	38 ± 4
LVEDV (mL) *	113 ± 18 (71–145)	46–121	30%	113 ± 18	116 ± 14	106 ± 22
LVEDV index (mL/m^2^) *	62 ± 9 (42–75)	20–70	15%	62 ± 9	63 ± 7	60 ± 11
LV Mass (g)	129 ± 24 (91–185)	51–173	6%	138 ± 24	125 ± 18	126 ± 31
LV mass index (g/m^2^) *	71 ± 12 (54–106)	33–99	3%	76 ± 11	68 ± 9	71 ± 16
LVEDV index(mL/(m^2^)^1.5^) *	62 ± 9 (42–76)	n/a	n/a	46 ± 7	46 ± 6	45 ± 8
Mean wall thickness (mm) *	7 ± 1 (6–9)	6–12	0%	7.6 ± 0.6	7.4 ± 0.5	7.5 ± 0.3
Relative wall thickness *	0.30 ± 0.03 (0.23–0.36)	≤0.42	0%	0.30 ± 0.02	0.30 ± 0.03	0.30 ± 0.03

* Denotes main effect of sex. n/a indicates no normative data available for comparison.

**Table 5:** Left ventricular functional parameters.

*Parameter*	*Mean ± SD of All Sailors (Range)*	*BSE Normative Range*	*% of Athletes Outside Normative Range*	*IG (Mean ± SD)*	*PG* *(Mean ± SD)*	*MG* *(Mean ± SD)*
**Male (*n* = 37)**						
Ejection fraction (%) *	57 ± 4 (49–66)	55–74%	38%	57 ± 4 **^#^**	54 ± 2	58 ± 3 **^##^**
GLS (%) *	−18 ± 2 (−23 to −14)	n/a	35%	−18 ± 2	−17 ± 2	−19 ± 2 **^##^**
Mean mitral annular s’ (cm/s)	12 ± 2 (9–16)	≥6.4	0%	12 ± 2	11 ± 2	12 ± 1
Mean mitral annular e’ (cm/s)	16 ± 3 (11–22)	>8	0%	16 ± 3	15 ± 2	16 ± 2
Average E/e’	5 ± 1 (3–7)	<14	0%	6 ± 1	5 ± 1	5 ± 1
E velocity (m/s)	0.8 ± 0.1 (0.5–1.1)	>0.5	0%	0.9 ± 0.2	0.8 ± 0.2	0.8 ± 0.1
E/A ratio	2.0 ± 0.6 (1.3–4.0)	>0.9	0%	2 ± 1	2 ± 1	2 ± 1
**Female (*n* = 33)**						
Ejection fraction (%) *	58 ± 4 (50–67)	55–74%	9%	58 ± 5 **^#^**	56 ± 3	60 ± 3 **^##^**
GLS (%) *	−19 ± 2 (−22 to −15)	n/a	18%	−19 ± 2	−18 ± 2	−19 ± 2 **^##^**
Mean mitral annular s’ (cm/s)	10 ± 1 (8–14)	≥6.4	0%	11 ± 1	10 ± 2	11 ± 1
Mean mitral annular e’ (cm/s)	16 ± 2 (12–21)	>10	0%	16 ± 3	15 ± 2	16 ± 2
Average E/e’	5 ± 1 (4–10)	≤14	0%	6 ± 2	5 ± 1	5 ± 1
E velocity (m/s)	0.8 ± 0.1 (0.6–1.2)	>0.6	0%	0.9 ± 0.2	0.8 ± 0.1	0.9 ± 0.2
E/A ratio	2.0 ± 0.6 (1.3–3.8)	>1.1	0%	2 ± 1	2 ± 1	2 ± 1

* Denotes main effect of sex. **^#^** Denotes a significant difference (*p* < 0.05) between isometric and pumping groups. **^##^** Denotes a significant difference (*p* < 0.05) between pumping and movement groups. n/a indicates no normative data available for comparison.

**Table 6:** Right ventricular structural and functional parameters.

Parameter	Mean ± SD of All Sailors (Range)	BSE Normative Range	% of Athletes Outside Normative Range	IG (Mean ± SD)	PG (Mean ± SD)	MG (Mean ± SD)
**Male (*n* = 37)**						
RVOT1 PSAX (mm) *	31 ± 4 (20–41)	24–44	0%	32 ± 4	29 ± 4	31 ± 3
RVOT1 PSAX index (mm/(m^2^)^0.5^) *	21 ± 2 (14–26)	n/a	n/a	22 ± 3	20 ± 3	22 ± 2
RVD1 base (mm) *	43 ± 5 (34–53)	26–47	14%	44 ± 5	43 ± 4	44 ± 5
RVD1 base index (mm/(m^2^)^0.5^) *	29 ± 3 (23–37)	n/a	n/a	30 ± 4	29 ± 3	31 ± 3
RVD2 mid (mm) *	70 ± 10 (24–40)	19–42	0%	31 ± 4	33 ± 4	34 ± 4
RVD2 mid index (mm/(m^2^)^0.5^) *	22 ± 3 (17–28)	n/a	n/a	22 ± 3	23 ± 3	24 ± 3
RVED area index (cm^2^/m^2^) *	12.6 ± 2.2 (7.7–17.9)	≤13.6	22%	12.9 ± 2.7	11.9 ± 1.4	12.9 ± 2.1
RV FAC (%)	42 ± 8 (29–59)	≥30	5%	43 ± 10	40 ± 6	42 ± 7
RV free wall strain (%)	−25 ± 3 (−31 to −20)	n/a	21%	−26 ± 3	−25 ± 4	−24 ± 2
RV S’ (cm/s)	14 ± 2 (8–18)	≥9	0%	14 ± 3	13 ± 3	14 ± 2
TAPSE (mm)	23 ± 4 (16–32)	≥17	3%	24 ± 5	22 ± 2	23 ± 3
**Female (*n* = 33)**						
RVOT1 PSAX (mm) *	28 ± 4 (18–35)	20–42	0%	29 ± 5	27 ± 4	27 ± 5
RVOT1 PSAX index (mm/(m^2^)^0.5^) *	21 ± 2 (16–24)	n/a	n/a	21 ± 3	20 ± 3	21 ± 2
RVD1 base (mm) *	38 ± 4 (31–47)	22–43	9%	37 ± 4	39 ± 3	39 ± 4
RVD1 base index (mm/(m^2^)^0.5^) *	28 ± 3 (23–35)	n/a	n/a	28 ± 3	29 ± 3	28 ± 3
RVD2 mid (mm) *	28 ± 4 (22–39)	17–35	3%	27 ± 3	30 ± 4	27 ± 3
RVD2 mid index (mm/(m^2^)^0.5^) *	21 ± 3 (16–28)	n/a	n/a	20 ± 2	21 ± 2	21 ± 2
RVED area index (cm^2^/m^2^) *	11.3 ± 1.5 (8.8–14.6)	≤13.6	6%	11 ± 2	11 ± 1	11 ± 2
RV FAC (%)	43 ± 6 (33–57)	≥35	6%	42 ± 5	44 ± 6	44 ± 6
RV free wall strain (%)	−25 ± 4 (−32 to −20)	n/a	21%	−25 ± 2	−25 ± 3	−24 ± 4
RV S’ (cm/s)	13 ± 2 (10–15)	≥9	0%	13 ± 2	13 ± 2	13 ± 2
TAPSE (mm)	22 ± 3 (17–29)	≥17	0%	21 ± 2	21 ± 3	23 ± 5

* Denotes main effect of sex. n/a indicates no normative data available for comparison.

**Table 7:** Atrial structural and functional parameters.

Parameter	Mean ± SD of All Sailors (Range)	BSE Normative Range	% of Athletes Outside Normative Range	IG (Mean ± SD)	PG (Mean ± SD)	MG (Mean ± SD)
**Male (*n* = 37)**						
LA ESV (mL) *	56 ± 11 (38–85)	24–77	3%	60 ± 11	53 ± 10	57 ± 10
LA ESV index (mL/m^2^)	27 ± 5 (19–42)	≤38	3%	29 ± 6	25 ± 5	28 ± 5
LA ESV index (mm/(m^2^)^1.5^)	19 ± 4 (13–29)	n/a	n/a	20 ± 4	17 ± 4	20 ± 3
RA area (cm^2^) *	19 ± 3 (10–25)	≤22	16%	20 ± 3	18 ± 4	20 ± 2
RA area index (cm^2^/m^2^) *	9 ± 2 (5–12)	≤11	8%	10 ± 1	9 ± 2	10 ± 1
LA reservoir strain (%) *	34 ± 5 (26–45)	25–63%	0%	35 ± 6 **^#^**	32 ± 5	35 ± 4 **^##^**
LA conduit strain (%)	−25 ± 5 (−39 to −17)	n/a	n/a	−26 ± 6 **^#^**	−23 ± 3	−26 ± 4 **^##^**
LA pump strain (%) *	−9 ± 3 (−16 to −2)	−2 to −23%	0%	−9 ± 4	−9 ± 3	−9 ± 3
**Female (*n* = 33)**						
LA ESV (mL) *	46 ± 11 (29–78)	20–67	6%	45 ± 12	47 ± 8	47 ± 15
LA ESV index (mL/m^2^)	25 ± 6 (16–40)	≤38	3%	25 ± 12	25 ± 4	27 ± 7
LA ESV index (mm/(m^2^)^1.5^)	18 ± 5 (6–29)	n/a	n/a	18 ± 5	18 ± 5	20 ± 5
RA area (cm^2^) *	15 ± 2 (9–20)	≤19	6%	15 ± 3	15 ± 2	15 ± 2
RA area index (cm^2^/m^2^) *	8 ± 1 (5–11)	≤11	0%	8 ± 2	8 ± 2	9 ± 1
LA reservoir strain (%) *	38 ± 6 (27–48)	29–62%	3%	40 ± 5 **^#^**	35 ± 6	40 ± 5 **^##^**
LA conduit strain (%)	−27 ± 6 (−38 to −13)	n/a	n/a	−28 ± 5 **^#^**	−24 ± 6	−30 ± 4 **^##^**
LA pump strain (%) *	−11 ± 3 (−17 to −7)	−2 to −21%	0%	−12 ± 3	−11 ± 3	−10 ± 3

* Denotes main effect of sex. **^#^** Denotes a significant difference (*p* < 0.05) between isometric and pumping groups. **^##^** Denotes a significant difference (*p* < 0.05) between pumping and movement groups. n/a indicates no normative data available for comparison.

## Data Availability

The data presented in this study are available on request from the corresponding author.
